# Measuring disability and monitoring the UN Convention on the Rights of Persons with Disabilities: the work of the Washington Group on Disability Statistics

**DOI:** 10.1186/1471-2458-11-S4-S4

**Published:** 2011-05-31

**Authors:** Jennifer H Madans, Mitchell E Loeb, Barbara M Altman

**Affiliations:** 1 National Center for Health Statistics, Hyattsville, MD 20782, USA

## Abstract

The Washington Group on Disability Statistics is a voluntary working group made up of representatives of over 100 National Statistical Offices and international, non-governmental and disability organizations that was organized under the aegis of the United Nations Statistical Division. The purpose of the Washington Group is to deal with the challenge of disability definition and measurement in a way that is culturally neutral and reasonably standardized among the UN member states. The work, which began in 2001, took on added importance with the passage and ratification of the UN Convention on the Rights of Persons with Disabilities since the Convention includes a provision for monitoring whether those with and without disabilities have equal opportunities to participate in society and this will require the identification of persons with disabilities in each nation. The International Classification of Functioning, Disability and Health (ICF) developed by the World Health Organization provided a framework for conceptualizing disability. Operationalizing an ICF-based approach to disability has required the development of new measurement tools for use in both censuses and surveys. To date, a short set of six disability-related questions suitable for use in national censuses has been developed and adopted by the Washington Group and incorporated by the United Nations in their Principles and Recommendations for Population and Housing Censuses. A series of extended sets of questions is currently under development and some of the sets have been tested in several countries. The assistance of many National and International organizations has allowed for cognitive and field testing of the disability questionnaires in multiple languages and locations. This paper will describe the work of the Washington Group and explicate the applicability of its approach and the questions developed for monitoring the UN Convention on the Rights of Persons with Disabilities.

## Introduction

The UN Convention on the Rights of Persons with Disabilities [1] represents an international milestone acknowledging the shift in attitudes and approaches to persons with disabilities that have been evolving over the past few decades. The Convention recognizes that disability results from the interaction between persons with impairments and the barriers (both attitudinal and environmental) that hinder their full and effective participation in society on an equal basis with others.

The Convention is intended as a human rights instrument with an explicit social development dimension. It recognizes the broad diversity among persons with disabilities and reaffirms that all persons with all types of disabilities should enjoy every human right and fundamental freedom. More specifically, included among the Convention’s general principles are the full and effective participation and inclusion in society, non-discrimination, accessibility and equality of opportunity for people with disabilities.

The Convention also includes specific requirements (Article 33) that focus on the establishment of mechanisms that would ensure the implementation and monitoring of the Convention at the national level. Implementation and monitoring of the Convention will require the collection of data on the population with disabilities for the countries that have ratified the Convention. Considering the complexity of the disablement process [2] and the diversity in reported disability prevalence internationally [3,4], the International Classification of Functioning, Disability and Health (ICF) developed by the World Health Organization (WHO) [5] provides a common language and a common point of reference for conceptualizing disability. Operationalizing an ICF-based approach to disability has required the development of new measurement tools for use in censuses and surveys. The earlier impairment-based, medical model approach that focused on medical conditions and asked some variation of the question “Do you have a disability?” is no longer satisfactory; and the focus of measurement has shifted to experienced difficulties in basic actions, more complex activities and barriers to participation [6,7]. The Washington Group on Disability Statistics is working to provide internationally comparable data within the framework of the ICF Model that would fulfill the stipulated requirement to monitor the UN Convention on the Rights of Persons with Disabilities.

## The Washington Group on disability statistics

The need for comparable population-based measures of disability for individual country use and for international comparisons was recognized in June of 2001 at the United Nations International Seminar on the Measurement of Disability. This determination was based on the scarcity and general poor quality of data on disability, especially in developing countries, and the lack of internationally comparable measures, even among developed countries. The Washington Group on Disability Statistics was formed to address this urgent need.

The main purpose of the Washington Group is to promote and co-ordinate international co-operation in the area of health statistics focusing on disability measures suitable for censuses and national surveys. The major objective is to develop tools to collect the basic data necessary to provide information on disability that is comparable throughout the world. The first priority of the Washington Group was to guide the development of a short set of disability measures suitable for use in censuses, sample-based national surveys, or other statistical formats, for the primary purpose of informing policy on equalization of opportunities for the population with disabilities. A second priority was to recommend one or more extended sets of survey items that elaborate the measurement of the multiple concepts associated with disability for use as components of population surveys, as supplements to surveys or as the core of a disability survey. The extended sets of survey items will be connected to the short set of disability measures. The disability measures recommended by the group are accompanied by descriptions of their technical properties, as well as methodological guidance for their implementation and their applicability to all population subgroups.

To date, the Washington Group has met nine times, in: Washington DC, USA (2002); Ottawa, Canada (2003); Brussels, Belgium (2004); Bangkok, Thailand (2004); Rio de Janeiro, Brazil (2005); Kampala, Uganda (2006); Dublin, Ireland (2007), Manila, the Philippines (2008) and most recently in Dar es Salaam, Tanzania (2009). The 10th meeting will be held in Luxembourg in 2010. All National Statistical Offices are eligible for membership in the Washington Group. Currently, representatives from 116 National Statistical Offices have formally indicated their interest in participating in the Washington Group (82 have attended at least one annual meeting of the group), as well as representatives from other international organizations, organizations that represent persons with disabilities (DPOs), the United Nations Statistical Division (UNSD), and other UN affiliates. The Secretariat for the Washington Group is located at the National Center for Health Statistics, USA. Details of the Washington Group organization, history and accomplishments are available online at: http://www.cdc.gov/nchs/citygroup.htm. In addition, the site provides access to lists of participants, proceedings from the meetings (presentations and papers), reports to the UN Statistical Commission and information on upcoming meetings.

The Washington Group has also fostered international cooperation by working with the UNSD, WHO, UN Economic and Social Commission for Asia and the Pacific (UNESCAP), UN Economic and Social Commission for Western Asia, UN Economic Commission for Europe (UNECE), International Labor Organization , Organization for Economic Development and Co-operation, Inter-American Development Bank, World Bank, Eurostat, the Budapest Initiative, SINTEF (an independent Norwegian research institute), and others to promote a unified approach to disability measurement. Several World Bank data instruments have been heavily influenced by the work of the Washington Group (in India and Uzbekistan), and SINTEF has been working in Africa to conduct independent tests of the Washington Group questions. In addition, the UNESCAP and WHO, in partnership with the Australian Bureau of Statistics, conducted pilot studies on the Washington Group short set of questions and the longer set of WHO questions. The study results were presented and discussed during the fifth and sixth Washington Group meetings. The Washington Group has also been informed that the question set has been pre-tested or added to surveys in at least 11 countries. The Group has embarked upon a collaboration with UNESCAP for the cognitive and field testing of the first extended set of questions in six participating UNESCAP countries. Cognitive testing is also taking place in Europe through the Granada Group. Finally, the Washington Group continues to dialogue with the World Bank in matters of common interest and in attempts to secure funding for further activities in the testing and development of extended sets of questions on disability in other regions.

The Washington Group has built capacity for disability data collection in developing countries by training government statisticians on disability measurement methodology. Regional training meetings held in Kenya (June, 2005) and Brazil (September, 2005) were an integral part of this effort. Presently, countries that received training are working internally to improve their overall approaches to dealing with the issue of disability measurement through ongoing data collection activities. Other capacity building and training activities have included:

a) UNECE Workshop on Disability Statistics in Special Programme for the Economies of Central Asia (SPECA) member countries (Bishkek, Kyrgyz Republic, 13-15 December 2006). The aim of the Training Workshop was to introduce participants to the best practices on Disability Statistics and to develop expertise in methodologies of measurement of the health status of the population. Health statistics directors and staff engaged in the measurement of disabilities in the national statistical offices and ministries of health in Central Asia and Azerbaijan attended.

b) Joint UNECE-UNFPA (United Nations Population Fund) Training Workshop on Census Management in South East Europe (Sarajevo, Bosnia and Herzegovina, 18-22 February 2008).

c) Workshop on Strengthening Capacity for Disability Measurement across South Asia sponsored by the World Bank and a Regional Workshop on Promoting Disability Data Collection through the 2010 Population and Housing Censuses sponsored by the United Nations in Bangkok, Thailand (April, 2008).

d) Joint UNECE-UNFPA Regional Training Workshop on Population and Housing Censuses for South Eastern European countries held in Ohrid, Macedonia (November 2008). The workshop was organized for senior professionals/experts from the State Statistical Offices of Albania, Bosnia and Herzegovina, Bulgaria, Croatia, Kosovo, Macedonia, Montenegro, Romania, and Serbia. The Washington Group was responsible for a full day training session including the measurement of disability in censuses and interpreting and understanding disability as measured using the Washington Group short set of questions.

e) In August/September 2009, at the request of the World Bank, the Washington Group assisted the Bangladesh Bureau of Statistics through a training workshop designed to provide an understanding of disability and functioning using the ICF-based Washington Group approach, and implementing the Washington Group short set of questions in their national Household Income and Expenditure Survey and in preparation for the 2010 census.

In addition to funding countries to conduct the tests, the Washington Group used a World Bank grant to employ a consultant from January to June 2006 to provide technical training and to support national statistics offices engaged in test activities. In-person technical support was provided to two African national statistical offices. Assistance via phone and email was provided to countries in Africa and South America as well as Viet Nam, the Philippines, and India.

## Measuring disability

Disability represents a complex process and is not a single, static state. It refers to the outcome of the interaction of a person and his/her environment (physical, social, cultural or legislative) and represents a measure of the negative impact of environmental factors on one’s ability to participate. The complexity of the concept has resulted in the proliferation of statistics on disability that are neither comparable nor easy to interpret. Furthermore, disability data are collected for different purposes such as to estimate the prevalence of physical impairments or to plan for the provision of services. Each purpose elicits a different statistic and even when the intention is to measure the same concept, the actual questions used differ in ways that severely limit comparability. The conclusion is not that some estimates are right and others are wrong, but that they are measuring different things. The Washington Group chose to develop questions that would address the issue of whether persons with disability participate to the same extent as persons without disabilities in activities such as education, employment or family/civic life. A major reason for this choice is the pivotal importance of the issue of social participation and equal rights from a policy perspective as illustrated in the UN Convention on the Rights of Persons with Disabilities [1]. In addition, there was agreement that it would be possible to develop a question set to meet this objective that could be administered using Census methodology and that could produce internationally comparable data.

One approach to measuring social engagement is to ask directly if a disability has impacted participation. An example of such a question is “Are you limited in the kind or amount of activities that you can do because of on-going difficulties due to long term physical, mental or health problems?” Such questions are difficult to ask in a way that produces comparable data. An alternative approach is to obtain information on difficulty in functioning in basic actions (see below) since these actions form the building blocks for more complex activities and, in an unaccommodating environment, can result in disparities in participation. The task is then to determine whether persons with difficulties or limitations in basic actions have participation rates equal to those without these limitations. It is also much easier to obtain comparable data on these universal functions.

The Washington Group questions were designed to provide comparable data cross-nationally for populations living in a variety of cultures with varying economic resources. While the ideal would be to collect information on all aspects of the disablement process and to identify every person with a disability within every community, this would not be possible given the limited number of questions that can be asked on a National Census. In unfriendly environments, the basic actions represented in this question set are those that are most often result in participation restrictions. Domains were selected using the criteria of simplicity, brevity, universality and comparability. It is expected that the information that results from the use of these questions will: a) represent the majority of, but not all, persons with limitation in basic actions; b) represent the most commonly occurring limitations in basic actions; and c) be able to capture persons with similar problems across countries.

## The Washington Group short set

The Washington Group developed a short set of questions for use in censuses and surveys according to the Fundamental Principles of Official Statistics [8] and which is consistent with the ICF. These questions were developed for administration using Census methodology and testing has shown that they produce internationally comparable data [9].

It is intended that these questions will identify the majority of persons in the population who are at greater risk than the general population of experiencing limited or restricted participation in society. The questions cover six functional domains or basic actions: seeing, hearing, walking, cognition, self care, and communication. The short question set reads as follows:

“The next questions ask about difficulties you may have doing certain activities because of a HEALTH PROBLEM” (see Table 1).

**Table 1:** Health Problem

		No difficulty	Some difficulty	A lot of difficulty	Cannot do it at all
**a**	Do you have difficulty seeing, even if wearing glasses?	1	2	3	4
**b**	Do you have difficulty hearing, even if using a hearing aid?	1	2	3	4
**c**	Do you have difficulty walking or climbing steps?	1	2	3	4
**d**	Do you have difficulty remembering or concentrating?	1	2	3	4
**e**	Do you have difficulty (with self-care such as) washing all over or dressing?	1	2	3	4
**f**	Using your usual (customary) language, do you have difficulty communicating, (for example understanding or being understood by others)?	1	2	3	4

Each question has four response categories: (1) No, no difficulty, (2) Yes, some difficulty, (3) Yes, a lot of difficulty and (4) Cannot do it at all. The severity scale is used in the response categories in order to capture the full spectrum of functioning from mild to severe. Multiple disability scenarios can be described depending on the domain(s) of interest and the choice of severity cut-off. There is more than one way to capture disability through the application of this set of core questions; resulting in not one but several possible prevalence estimates.

The focus on measuring functioning in core domains is in contrast to approaches that are based on impairments or deviations or loss in various bodily structures. A more detailed discussion of the conceptual framework and data collection objectives of the Washington Group can be found in the Washington Group Position Paper: Proposed Purpose of an Internationally Comparable General Disability Measure [6].

The approach to disability measurement taken by Washington Group has also been incorporated into the UN Principles and Recommendations for Population and Housing Censuses [10] (See: Section VI-8: Disability Characteristics pages 178-183, and Tabulations on Disability Characteristics pages 292-294). Over 50 countries on all continents have been involved in the testing of the Washington Group short set or have used the questions in their national data collections. Twenty countries have announced their intent to use the questions on their Censuses; many others have not made final decisions and yet others will add the questions to other national surveys.

## Data to monitor the UN Convention

Ratification and endorsement of the UN Convention on the Rights of Persons with Disabilities are the initial steps to establishing awareness and compliance at the national level. The United Nations has also requested that means be sought to develop a set of indicators intended to monitor the implementation of the Convention. This proposal is closely related to the work of the Washington Group. The same tools (short set and extended questions) developed as measures of equalization of opportunity under the aegis of the Washington Group would service equally to monitor the UN Convention.

The Washington Group chose to develop questions that would address a specific aspect of the disablement process, the issue of whether persons with disability participate to the same extent as persons without disabilities in activities such as education, employment or family/civic life; in other words, the equalization of opportunities, which is one of the general principles listed in Article 3 of the Convention and the focus of Article 5 (Equality and Non-discrimination). It is also particularly relevant to the collection of data for policy purposes outlined in Article 31 (Statistics and data collection) and will facilitate the monitoring of participation in cultural life, leisure, and recreation (Article 30), and work & employment (Article 27) [1].

The Washington Group short set of six questions, when incorporated in Censuses or surveys, can provide baseline information that can fulfill the requirements for monitoring. By using these standard questions it will be possible to provide comparable data cross-nationally for populations living in a variety of cultures with varying economic resources; comparable data that can be used to assess a country’s compliance with the UN Convention and, over time, their improvement in meeting the requirements set out under the Convention. The recommended short set of questions will identify the majority of the population with difficulties in functioning in basic actions; difficulties that have the potential to limit independent living or social integration if appropriate accommodation is not made.

This indicator, coupled with other information collected through the Census or survey on complex activities, for example, employment, education, or family & social life, can then be used to compare the levels of participation in these complex activities between those with disability (as measured by difficulty in performing basic actions) and those without – and thereby assess equitable access to opportunities as mandated by the UN Convention. For example, data on difficulty in performing basic actions can be cross-classified with a measure of employment to identify the proportion of persons with and without disability who are employed. This is an assessment of the equality of employment opportunities. If policy interventions are initiated to enhance workplace accommodations, the effect on employment of persons with disability can be determined by comparing baseline data to data collected after the policy changes have been implemented. In addition to employment, it will be important to collect data on a variety of forms of participation, such as education, housing, transportation, social and health services, in addition to aspects of family, cultural and social life. From a theoretical perspective, if opportunities have been optimized then participation should be equal between persons with and without disability. A trend analysis would also show improvements among those with disabilities over a period of time.

In addition to the short set, and given the determination of disability, further information could be collected on the mechanisms that facilitate or impede participation in complex activities, such as environmental and attitudinal barriers to equitable access. Environmental barriers or facilitators can exist on several levels:

• micro-environment: defined in terms of personal and technical assistance (that which follows the person wherever they go, for example wheelchair, glasses, or personal attendant);

• meso-environment: refers to the environment beyond the person (accessibility is facilitated or hindered based on, for example, transportation infrastructure, service provision at the local level, or attitudes of others);

• macro-environment: refers to affects on a regional, societal or national scale, such as policies, legislation, or general societal attitudes and practices.

**Figure 1:**
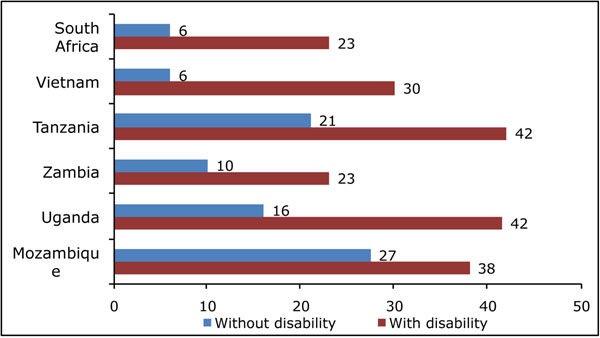
**Population aged 15 years + who never attended school, by disability status (%)**.

Figure 1 represents data that have currently been collected internationally using the Washington Group short set of questions, and that can be used to monitor the Convention; in this case, in terms of school attendance. For this analysis, disability has been determined as those who report having at least some difficulty doing at least two of the six actions included (this would also include those who report a lot of difficulty or who were unable to do any one of those actions).

Each of the countries included in the analysis report significant discrepancies in school attendance by those with and without disability; and these data then form the baseline for follow-up studies that would monitor the countries’ implementation of policies and actions that would eventually reduce these discrepancies.

Table 2 is a depiction of what a follow-up analysis might present. New data collected (say in 2012) could be compared to baseline data from earlier surveys (2006/2008) and progress (or lack thereof) could be monitored. This type of analysis – based on disability measurement analyzed in conjunction with other data on education, employment and social participation – would meet the requirements of the UN Convention to monitor its implementation.

**Table 2:** Proposed table shell illustrating comparison of percent with any education from baseline (2006/2008) to future (2012) levels by disability status.

	Year of survey 2006/2008			Follow-up Survey 2012			
		
Country	With disability	Without disability	Difference	With disability	Without disability	Difference	Change in Difference
Mozambique	38	27					
Uganda	42	16					
Zambia	23	10					
Tanzania	42	21					
Vietnam	30	6					
South Africa	23	6					

## Extended question sets: next Washington Group product

The Washington Group acknowledges that the six questions (short set) do not capture all people at risk of experiencing the disadvantage associated with disability, and has therefore embarked upon the development of extended sets of questions. These sets, intended as components of population surveys, as supplements to surveys, or as the core of a disability survey, go into greater depth on the same 6 domains covered by the short set of questions – including questions on functioning with and without assistance or assistive technologies, the age at onset and the impact of the difficulty on peoples’ lives. The extended set of questions also includes more domains such as learning, affect, pain and fatigue.

**Figure 2:**
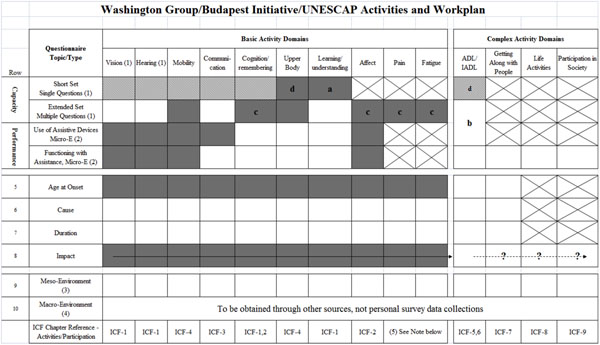
**Matrix developed to guide the development of the extended sets of questions**. 1. Measurement is WITHOUT the use of assistive devices or other help WITH THE EXCEPTION OF VISION (glasses/lenses) and HEARING (hearing aids). These are both measured WITH the use of assistive devices and thus do NOT represent true measures of Capacity. As such, extended set multiple questions are captured under Performance (row 4). NB - SEVERITY is captured in response categories. 2. Micro-environment: technical and personal assistance that follows the persons wherever they go (e.g. wheelchair, eye glasses, personal attendant). ICF Environment Chapter 1 & 3. 3. Meso-environment: the environment beyond the person (e.g. transportation infrastructure, accessibility, service provision at local level, attitudes of others). ICF Environment Chapters 2 & 4. Meso-environmental questions may also be non-domain specific. 4. Macro-environment: that which affects a whole country, such as policies and legislation, general societal attitudes and practices. ICF Environment Chapter 5. Macro-environmental questions are NOT domain specific. 5. Pain and Fatigue are not obvious functional domains (nor are they in the ICF); however, they are included here as domains. a) one question for children/one question for adults b) available for special populations c) no mention of functioning without AD - includes Intensity (How often?) d) upper body short set question is the ADL short set question

The matrix in Figure 2 depicts the extended set modules being developed – hash marks represent the existing short set of six questions, added columns represent additional domains, and rows depict the different aspects of functioning within those domains. Cells shaded in grey represent the set of extended questions tested in the UNESCAP region.

## Testing the first Washington Group set of extended questions

The UNESCAP has undertaken a set of projects to improve disability statistics in the Asia/Pacific region. The first project (2004 – 2006) focused on Improving Disability Statistics and Measurement, introduced the ICF as a framework for the development of questions on disability and functioning, discussed question design and testing (cognitive and field testing), and produced a Disability Statistics Training Manual. The current project (2008 – 2010), Improvement of Disability Measurement and Statistics in Support of Biwako Millennium Framework and Regional Census Programme, is a follow up to the earlier project and focuses on the cognitive and field testing of an extended set of disability questions. Participating countries include Cambodia, Kazakhstan, Maldives, Mongolia, Philippines, and Sri Lanka.

The aim of the project is to further promote better disability data collection by developing standard measurement tools, assessing and ensuring cross-national comparability, and improving national technical capacity. The project takes into account individual country needs in the region while contributing to the ongoing global initiatives on disability statistics. Its focus is on designing standard question sets for surveys, and conducting pilot tests and post-pilot test data analyses, thus providing an empirical basis for establishing standard survey measurement for disability data collection. The Washington Group is an active partner on the project that will add substantially to the development of comparable disability measures for international use.

## European testing and beyond

Cognitive testing of a modified extended set of questions is currently underway in Europe in association with the Budapest Initiative and the Granada Group, which includes France, Italy, Portugal, Spain, Germany and Switzerland. In addition, Canada, South Africa and the United States have also conducted cognitive interviews of the extended set of questions. The United States has added a module including the extended set of questions to the National Health Interview Survey.

Results from the analyses of data from these sources are expected by the end of 2010.

Finally, a second extended set of questions is scheduled for discussion at the 10th meeting of the Washington Group in Luxembourg in November, 2010. These questions will embrace issues of concerning the environment – more specifically environmental barriers and facilitators to participation.

## Conclusions

The Washington Group on Disability Statistics is a collaborative effort that involves countries at all levels of development from all corners of the globe. The intent of the group is to provide data on all aspects of the disablement process that is consistent with the framework provided by WHO in the form of the ICF. A short set of questions has been developed and intensively tested in many countries. This short set provides a comparable mechanism for identifying a population at risk for limitations in the ability to fully participate in society due to functional limitations in core domains. By monitoring the participation status of this population, it will be possible to determine if the objectives of the UN Convention on the Rights of Persons with Disabilities have been achieved.

## Competing interests

The authors declare that they have no competing interests.
